# Global research trajectories in gut microbiota and functional constipation: a bibliometric and visualization study

**DOI:** 10.3389/fmicb.2024.1513723

**Published:** 2024-12-06

**Authors:** Shun Seng Ong, Lianjie Xu, Ching Wei Ang, Xiaoyue Deng, Hai Lu, Tianshu Xu

**Affiliations:** ^1^Department of Traditional Chinese Medicine, Nanjing Drum Tower Hospital, The Drum Tower Clinical Medical College, Nanjing University of Chinese Medicine, Nanjing, China; ^2^First Clinical Medical College, Nanjing University of Chinese Medicine, Nanjing, China; ^3^Department of Traditional Chinese Medicine, Nanjing Drum Tower Hospital, Affiliated Hospital of Medical School, Nanjing University, Nanjing, China

**Keywords:** gut microbiota, functional constipation, bibliometric analysis, hotspots, trends

## Abstract

**Background:**

Functional constipation (FC) negatively impacts quality of life and is associated with gut microbiota (GM) imbalances. Despite the growing interest in this area, a thorough analysis of research trends is missing. This study uses bibliometric methods to assess the global research on GM’s role in FC, pinpointing key topics, impactful studies, and prominent researchers to guide future research and identify gaps.

**Methods:**

In our study, we conducted a performance analysis and science mapping using bibliometric indicators such as publication trends, author and institutional contributions, productivity, impact, keyword analysis, and collaboration networks. We employed software tools like VOSviewer, Biblioshiny, CiteSpace, and SCImago Graphica to automate the assessment of metrics including country, institutional, and journal distribution, authorship, keyword frequency, and citation patterns.

**Results:**

From 2013 to 2024, annual publications on GM and FC rose from 29 to 252, with a slight decrease to 192 in 2024. Average citations per publication peaked at 11.12 in 2021, declining to 6.43 by 2024. China led in research output (37.8%), followed by the United States (14.4%) and Japan (7.5%). Bibliometric analysis identified key authors like CHEN W and ZHANG H, with 30 and 27 articles, respectively. Jiangnan University and Harvard University were top contributors, with 131 and 81 articles. Keywords analysis revealed “constipation,” “gut microbiota,” and “probiotic” as central themes, with a shift toward “gut microbiota” and “intestinal flora” in recent years. This study provides a comprehensive overview of the research landscape, highlighting leading authors, institutions, and evolving research priorities in the field.

**Conclusion:**

Our review synthesizes current GM and FC research, guiding future studies. It suggests exploring GM in various GI disorders, the impact of lifestyle and drugs on GM, advanced research techniques, and probiotics/prebiotics for FC. There’s also a focus on therapies targeting GM’s effect on the gut-brain axis, paving the way for improved FC management.

## 1 Introduction

Functional constipation (FC) is a prevalent gastrointestinal disorder characterized by infrequent bowel movements, excessive stool hardness, and difficulty in defecation, which can significantly impair an individual’s quality of life (Barberio et al., 2021; Aziz et al., 2020). It is a complex condition that often emerges during critical periods of life, such as infancy, toilet training, and school entry (Barbara et al., 2023). The pathophysiology of FC is multifactorial, involving a combination of psychological, physiological, and dietary factors (Chang et al., 2023; Kilgore and Khlevner, 2024). Recent research has highlighted the potential role of gut microbiota (GM) in the regulation of gut motility and the development of FC (Lai et al., 2023; Ibarra et al., 2018; Strandwitz, 2018).

The GM, an intricate community of microorganisms residing in the gastrointestinal tract, is now recognized as a key player in gut health and disease (Fan and Pedersen, 2021; Ling et al., 2022). It is involved in various functions, including the fermentation of undigested food, synthesis of vitamins, and modulation of the immune system (Zmora et al., 2019; Fan et al., 2023). Dysbiosis, or an imbalance in the GM, has been associated with the pathogenesis of FC, suggesting that microbial imbalances might contribute to the symptoms of constipation (Weiss and Hennet, 2017; Cao et al., 2017).

Despite the growing interest in the role of GM in FC, a comprehensive understanding of the global research trends and scholarly communication in this field is lacking. Bibliometric analysis, a research method that uses quantitative techniques to analyze scientific literature, offers a systematic approach to mapping the intellectual structure of a field and identifying emerging research trends (Aria and Cuccurullo, 2017).

In this study, we employ bibliometric and visualization methods to provide a detailed overview of the global research landscape concerning GM and FC. By examining a broad spectrum of scientific publications, we aim to uncover the historical development, current status, and future directions of this research domain. Our analysis will identify the most influential studies, prolific authors, and leading institutions contributing to this field, as well as the key themes and concepts that have shaped the discourse.

Through a systematic mapping of the literature, we seek to answer several critical questions: What are the predominant research themes within the study of GM and FC? Which countries and institutions are leading the research efforts? What are the most impactful publications, and what do they contribute to our understanding of the field? By addressing these questions, we aim to provide a roadmap for future research and highlight potential gaps in the current body of knowledge.

This study contributes to the field by offering a macroscopic view of the research trends and hotspots in GM and FC, facilitating a more targeted and strategic approach to future research endeavors.

## 2 Materials and methods

### 2.1 Data sources and search strategy

**Figure 1:**
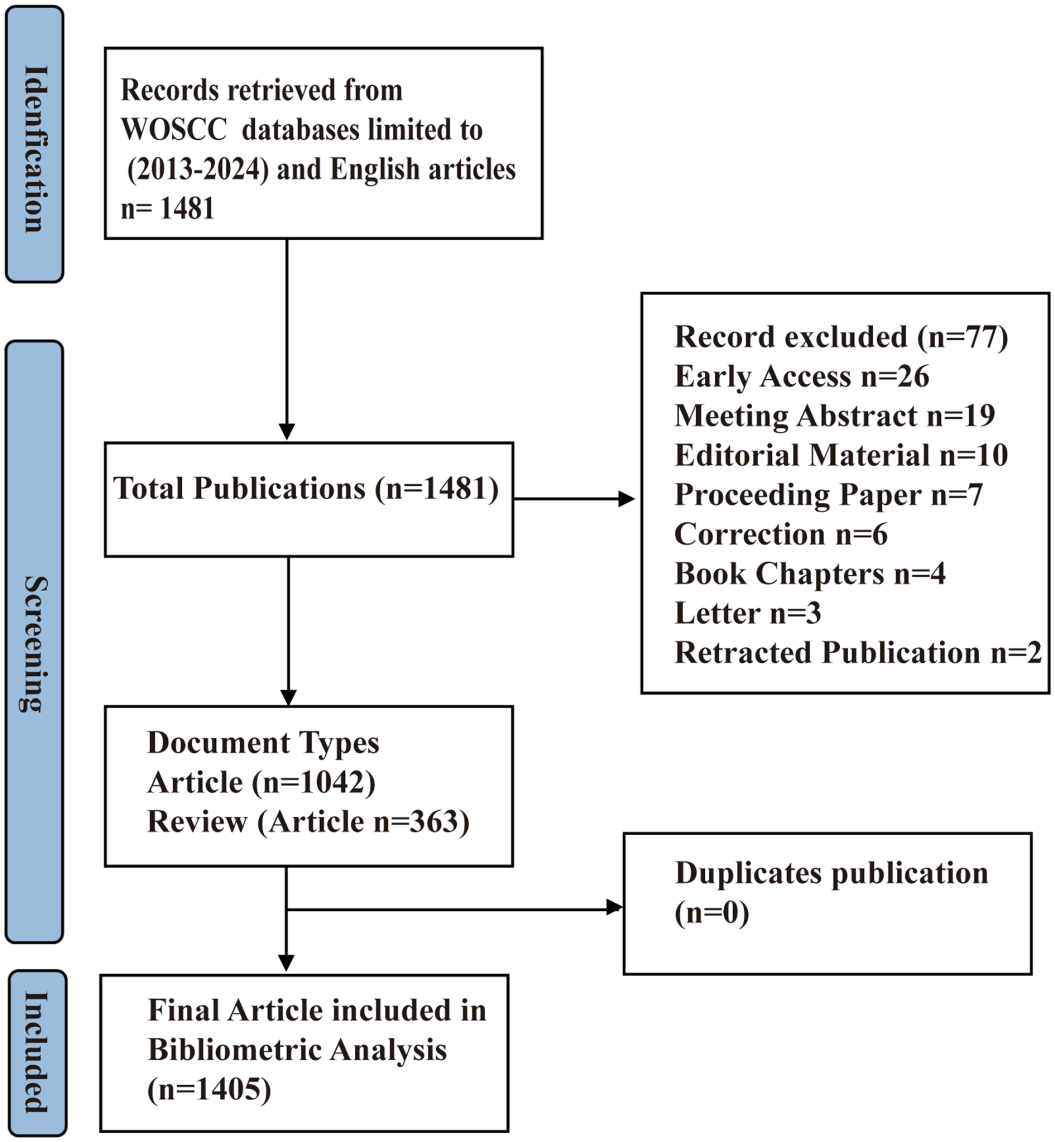
Flow chart of search strategy.

This bibliometric analysis was conducted following a systematic approach inspired by the PRISMA principles, tailored to accommodate the quantitative nature of our study. For this study, a comprehensive literature search was performed in the Web of Science Core Collection (WoSCC) database on September 30, 2024. The aim was to ensure a high level of reliability and representativeness in the selection of pertinent literature. To achieve this, we utilized Medical Subject Headings (MeSH) terms from PubMed, recognized for their specificity and sensitivity in literature retrieval, as the foundation for our search keywords The search strategy was meticulously constructed to encompass a wide range of terms related to GM, coupled with terms relevant to FC, as detailed below: *TS = (“Gastrointestinal Microbiomes” OR “Microbiome, Gastrointestinal” OR “Gastrointestinal Microbial Community” OR “Gastrointestinal Microbial Communities” OR “Microbial Community, Gastrointestinal” OR “Gut Microbiome” OR “Gut Microbiomes” OR “Microbiome, Gut” OR “Gut Microflora” OR “Microflora, Gut” OR “Gastrointestinal Microflora” OR “Microflora, Gastrointestinal” OR “Gastrointestinal Flora” OR “Flora, Gastrointestinal” OR “Gut Flora” OR “Flora, Gut” OR “Gastrointestinal Microbiota” OR “Gastrointestinal Microbiotas” OR “Microbiota, Gastrointestinal” OR “Gut Microbiota” OR “Gut Microbiotas” OR “Microbiota, Gut” OR “Intestinal Microbiome” OR “Intestinal Microbiomes” OR “Microbiome, Intestinal” OR “Intestinal Flora” OR “Flora, Intestinal” OR “Intestinal Microbiota” OR “Intestinal Microbiotas” OR “Microbiota, Intestinal” OR “Intestinal Microflora” OR “Microflora, Intestinal” OR “Enteric Bacteria” OR “Bacteria, Enteric” OR “Gastric Microbiome” OR “Gastric Microbiomes”) AND TS = (“functional constipation” OR “constipation” OR “Colonic Inertia” OR “Dyschezia”).* Our literature search was limited to English-language articles and reviews to maintain consistency and relevance. Any selection discrepancies were addressed through discussions between two researchers, with a third party arbitrating in cases of disagreement. A flowchart detailing the specific process of our search strategy is presented in Figure 1 and Supplementary Table S1.

### 2.2 Data analysis

Bibliometric analysis was conducted by focusing on two main components: performance analysis and science mapping. Performance analysis evaluated the research impact, considering metrics such as total publications, author contributions, and citation counts to assess the influence of individual researchers, institutions, and countries. Science mapping was utilized to visualize the structure and dynamics of scientific research, examining relationships between various research elements. Techniques such as citation analysis, co-citation analysis, bibliographic coupling, co-word analysis, and co-authorship analysis were employed.

In this study, we performed performance analysis and science mapping using bibliometric indicators, including publication trends, author and institutional contributions, productivity and impact of authors and journals, keyword analysis, and collaboration networks. Once the bibliometric methods were selected, we executed the analysis using appropriate software tools.

Data extracted from selected publications were analyzed utilizing VOSviewer 1.6.20, Biblioshiny (a Bibliometrix web interface), CiteSpace 6.3.R1 (64-bit), and SCImago Graphica. These software tools facilitated an automated assessment of key metrics, including country, institutional, and journal distribution, as well as authorship, keyword frequency, and citation patterns.

## 3 Results

### 3.1 Analysis of yearly publication trends and average citations

**Figure 2:**
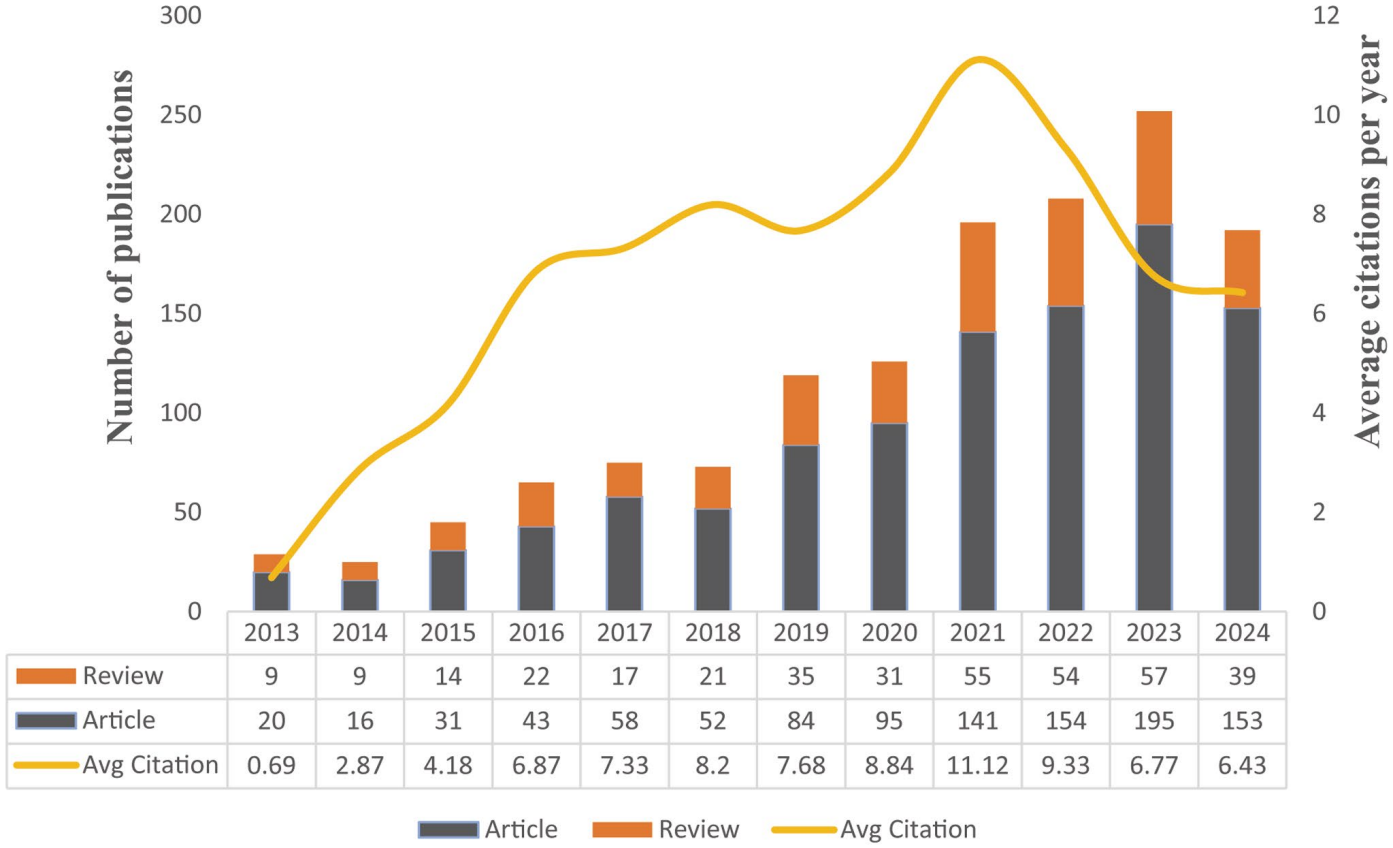
Analysis of publication volume and annual citation metrics for GM research in FC (2013–2024).

The longitudinal dataset spanning 2013–2024 delineates a consistent ascent in the annual publication figures, commencing with a total of 29 in 2013 and culminating in 252 in 2023, prior to a minor retracement to 192 in 2024 (Figure 2). Concurrently, the annual average citations per publication demonstrated a notable escalation, achieving a zenith of 11.12 in 2021, followed by a subsequent descent to 6.43 in 2024. This trajectory suggests a proliferation of research endeavors and an oscillating scholarly influence on research of GM and FC, with the latter years potentially witnessing a diminution in the citation-based impact.

### 3.2 Geographical distribution and collaborative patterns of research

**Figure 3:**
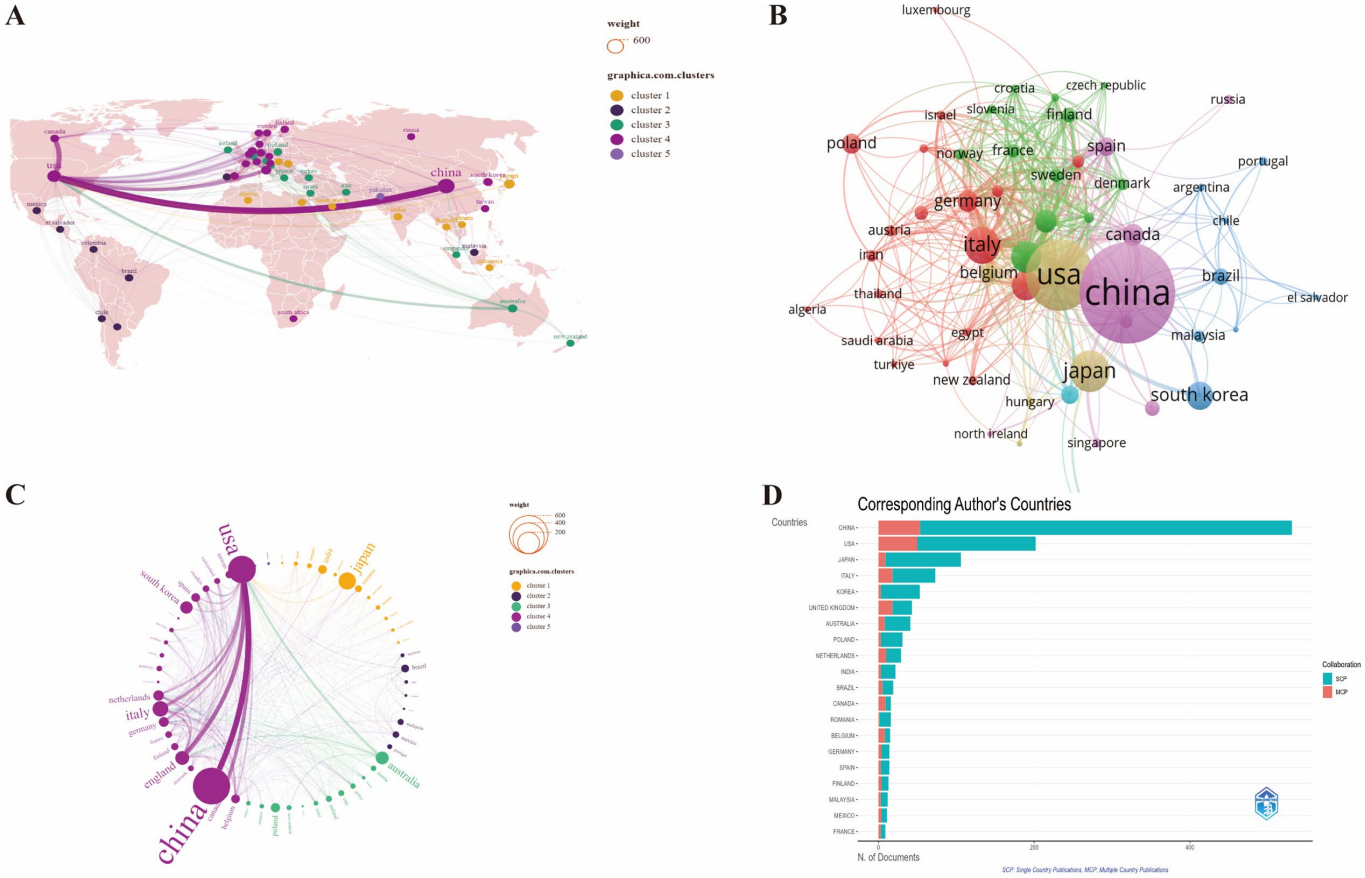
Visualization analysis of the geographical distribution of research on the correlation between GM and FC. **(A)** Global distribution of research by country and region. **(B)** Mapping of co-authorship collaboration network among countries/regions. **(C)** International research collaboration by country/region. **(D)** Ranking of the top 20 countries by publication output of corresponding authors. SCP, single country publications; MCP, multiple country publications.

To explore the geographical distribution of research on the correlation between GM and FC, we utilized SCImago Graphica, VOSviewer, and the Biblioshiny web application for data visualization and analysis (Figure 3). The bibliometric analysis reveals the distribution and collaborative patterns of research contributions in the field of GM and FC from 2013 to 2024. China is identified as the most prolific contributor, with 531 articles accounting for 37.8% of the total global publications. This is followed by the United States with 202 articles (14.4%) and Japan with 106 articles (7.5%). These figures highlight the leading role of these nations in the research output within this scientific domain.

The visual representation on the map delineates research output by country, with darker shades indicating a higher number of articles. Countries engaged in cooperative efforts are grouped within the same color cluster. The thickness of the lines connecting these countries is indicative of the strength of their collaborative relationships; thicker lines denote stronger ties, while thinner lines suggest less frequent collaboration (Figures 3A–C).

In terms of single-country publications (SCP), China dominates with 478 SCP, indicating a substantial body of research that does not involve international collaboration. The United States and Japan follow with 152 and 97 SCP, respectively. However, when considering multiple-country publications (MCP), which signifies collaborative efforts, the USA shows a notable level of international cooperation with 50 MCP, compared to China’s 53 MCP, suggesting a more dispersed collaborative network (Figure 3D; Supplementary Table S2).

Italy, Korea, the United Kingdom, Australia, Poland, the Netherlands, and India also contribute significantly to the field, albeit at a lower scale, with their publication numbers ranging from 22 to 73. These countries collectively contribute to the rich tapestry of global research in this area.

### 3.3 Analysis of authorship publication trends and collaborative networks

**Figure 4:**
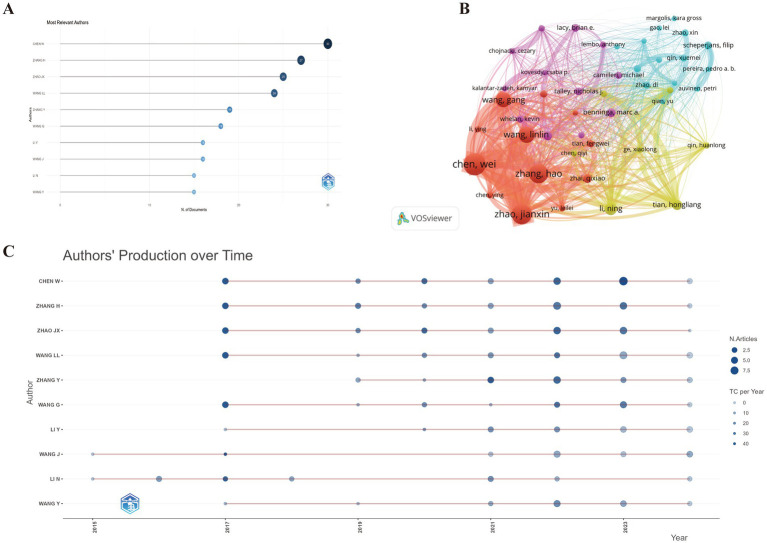
**(A)** Top 10 authors with the most significant contributions to research on the GM-FC correlation. **(B)** Collaboration network among countries/regions. **(C)** Yearly publication trends of the top 10 prolific authors in GM-FC correlation research.

A total of 7,409 authors participated in the discourse surrounding this field, encompassing 29 authors who authored single-authored documents and 7,359 authors who contributed to multi-authored documents. The observed average count of co authors per document stood at 7.29. The bibliometric analysis identifies CHEN W and ZHANG H as leading contributors to GM and FC research, with 30 and 27 articles, respectively (Figure 4A). CHEN W also dominates in total citations (863), followed by ZHAO JX (829) and ZHANG H (778). CHEN W’s H-index (15) is highest, tied with ZHAO JX, and exceeds ZHANG H’s (14). The M-index is highest for CHEN W and ZHAO JX (1.875), with ZHANG H close behind (1.75). CHEN W also leads in the G-index (29), followed by ZHANG H (27) and ZHAO JX (25), indicating a strong impact and extensive research influence (Supplementary Table S3).

Figure 4B illustrates the annual publication output of the top 10 authors and their collaboration network, highlighting a tight-knit cooperative group comprising CHEN W, ZHANG H, ZHAO JX, WANG G, and WANG LL. Figure 4C presents the annual citation count, where a darker shade corresponds to a greater total number of citations. The size of the circles is directly proportional to the authors’ publication volume, with the majority of the prolific authors’ works published between 2017 and 2023.

### 3.4 Analysis of institutional distribution and collaborative networks

**Figure 5:**
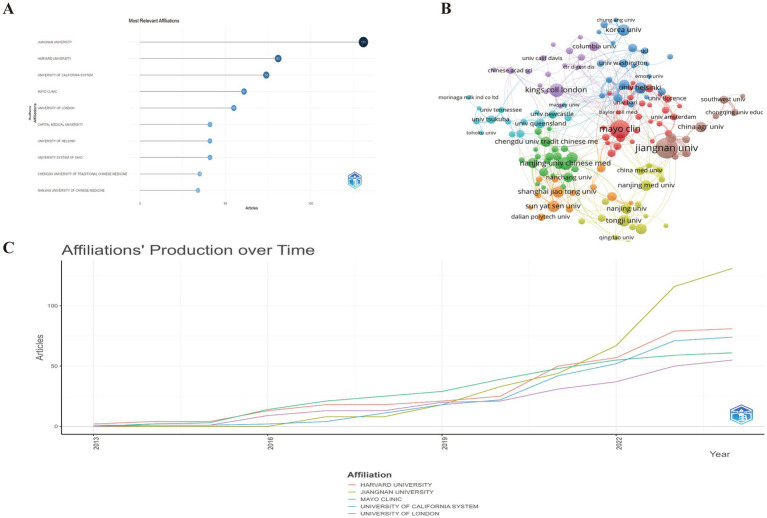
**(A)** Top 10 institution with the most significant contributions to research on the GM-FC correlation. **(B)** Collaboration network among institutions. **(C)** Yearly publication trends of the top 5 institutions in GM-FC correlation research.

Our analysis identified a total of 1,459 contributing institutions to the research domain of GM and FC. Prominently, Jiangnan University stands at the forefront with a significant publication count of 131 articles, underscoring its pivotal role. Harvard University follows in succession with a substantial contribution of 81 articles, reflecting its global research impact (Figure 5A; Supplementary Table S4). The University of California System secures the third position with 74 articles, indicating a robust and collaborative research environment.

The analysis focused on institutions contributing more than 5 papers to the field of GM and FC. As depicted in Figure 5B, these institutions are visualized in a network view that incorporates co-occurrence and cluster analyses, revealing eight distinct cooperative clusters. Within these clusters, institutions exhibit dense collaborative ties, and there is evidence of significant interaction across clusters as well.

Figure 5C highlights the recent publication activity of the top five affiliated institutions, showcasing their ongoing contributions to the research landscape.

### 3.5 Analysis of journals and cited journals

**Figure 6:**
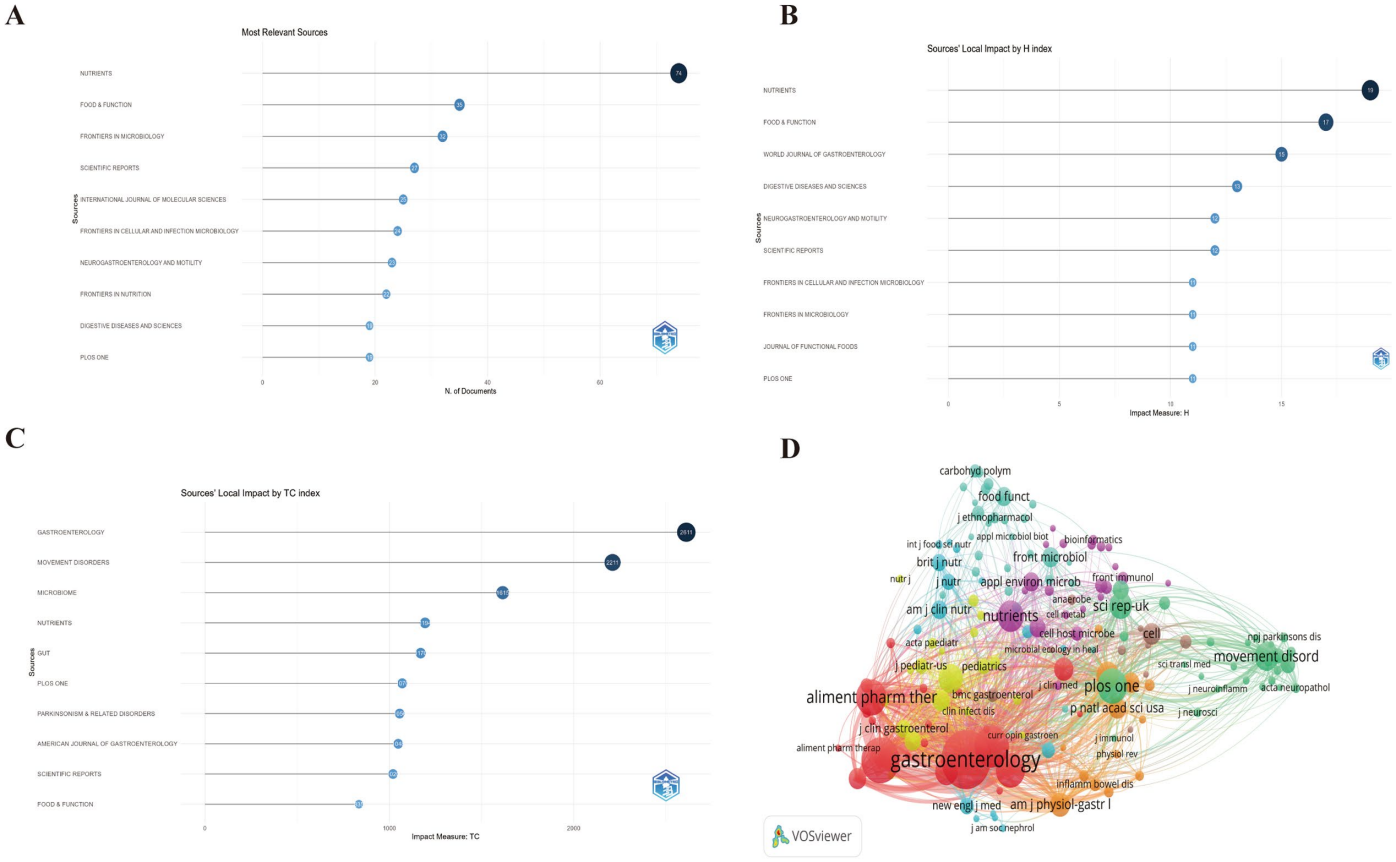
**(A)** Top 10 journals with the most significant contributions to research on the GM-FC correlation. **(B)** Top 10 journals by H-index influence. **(C)** Top 10 journals by total citation index. **(D)** Co-citation network of cited journals.

The analysis identified 486 distinct sources contributing to the field of GM and FC, with Nutrients publishing the highest number of relevant articles at 74, followed by Food and Function with 35, and Frontiers in Microbiology with 32 (Figure 6A; Supplementary Table S5). According to the H-index, the most influential journals are Nutrients (H-index of 19), Food and Function (H-index of 17), and the World Journal of Gastroenterology (H-index of 15) (Figure 6B). In terms of total citations, the leading journals are Gastroenterology with 2,611 citations, Movement Disorders with 2,211 citations, and Microbiome with 1,615 citations (Figure 6C; Supplementary Table S6). The co-citation network of these cited journals is depicted in Figure 6D.

### 3.6 Analysis of cited references

**Figure 7:**
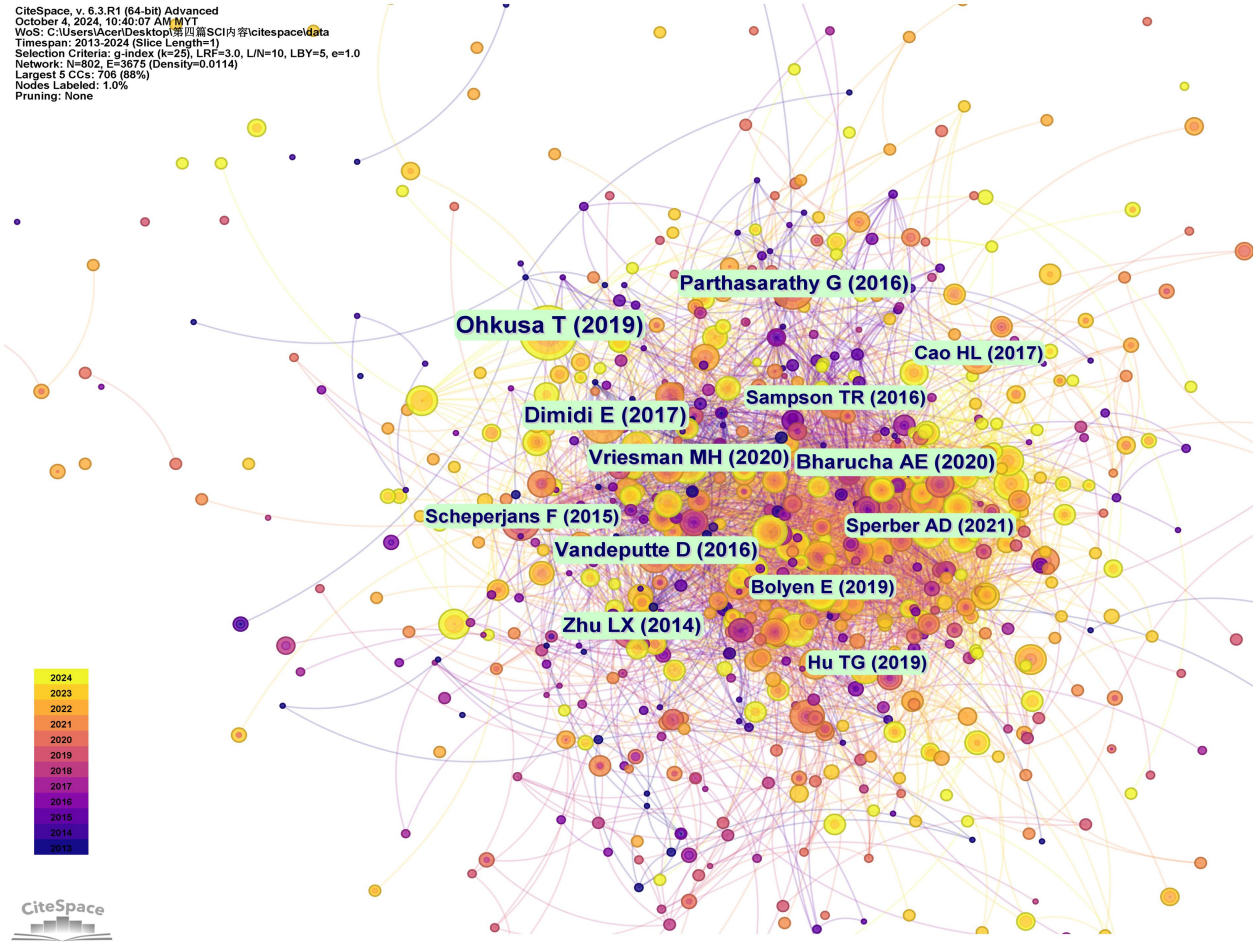
Network-visualization of cited references.

Co-citation relationships, which indicate the frequency with which multiple documents cite the same references, are instrumental in assessing the interconnectedness of various scholarly works. Prominently, the most cited work is Ohkusa T’s “Gut Microbiota and Chronic Constipation: A Review and Update,” published in Frontiers in Medicine in 2019. This is followed by Dimidi E’s “Mechanisms of Action of Probiotics and the Gastrointestinal Microbiota on Gut Motility and Constipation,” which appeared in Advances in Nutrition in 2017. The third most cited reference is Parthasarathy G’s study on the “Relationship Between Microbiota of the Colonic Mucosa vs. Feces and Symptoms, Colonic Transit, and Methane Production in Female Patients With Chronic Constipation,” featured in Gastroenterology in 2016 (Supplementary Table S7). The trio of studies collectively examines the link between chronic constipation and GM, revealing that distinct changes in fecal and mucosal microbiota correlate with the severity of constipation symptoms, colonic transit time, and levels of methane production (Ohkusa et al., 2019; Dimidi et al., 2017; Parthasarathy et al., 2016). These findings imply that targeted modulation of the GM may pave the way for novel therapeutic interventions for constipation. Figure 7 presents the network visualization of co-citation analysis.

### 3.7 Trends in keyword and research hotspot analysis

**Figure 8:**
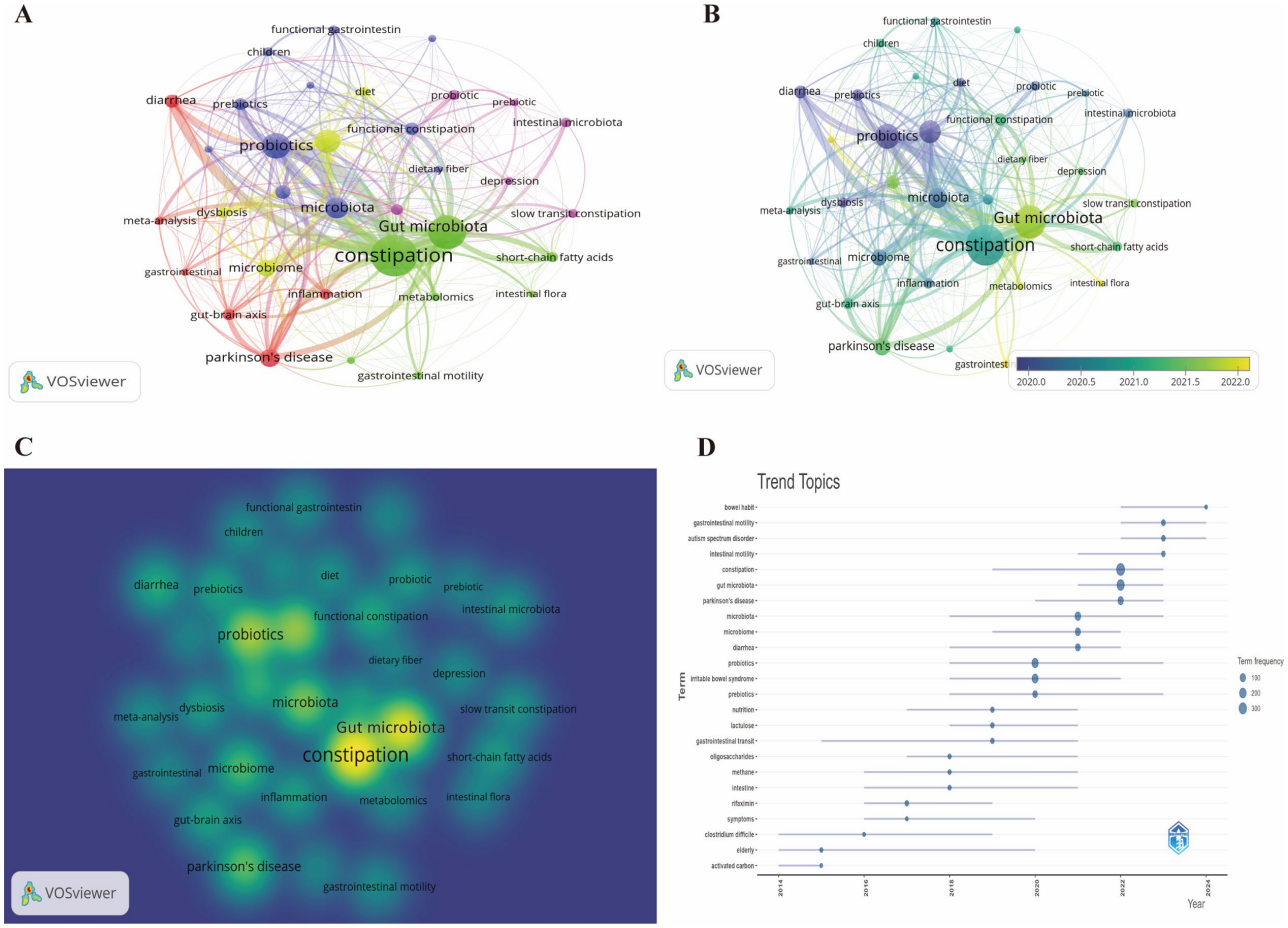
**(A)** Network visualization of keywords. **(B)** Overlay visualization of keyword network. **(C)** Density mapping of keyword network. **(D)** Trend topic analysis of keywords.

The analysis encompassed a total of 2,726 keywords, with a minimum occurrence threshold set at 20 to ensure the relevance and frequency of the included terms. The keyword network is visually represented in Figure 8A. The density visualization map was utilized to depict keywords occurring with similar frequencies (Figure 8C). The analysis revealed that the quintessential keywords associated with GM-FC research were “constipation” (370 occurrences), “gut microbiota” (234), “probiotic” (166), “irritable bowel syndrome” (143), and “microbiota” (117), thereby underscoring the prevailing research focal points (Supplementary Table S8).

Figure 8A illustrates the analysis, which categorized keywords into 5 distinct clusters, with the most prominent being the green, blue, and purple clusters. The green cluster from the keyword analysis is indicative of a prominent research theme, highlighting keywords like “constipation,” “enteric nervous system,” “gastrointestinal motility,” “gut microbiota,” “intestinal flora,” “metabolomics,” and “short-chain fatty acids.” These terms reflect a concentrated effort to understand the complex interplay between the gut’s microbial ecosystem and its physiological functions, particularly in the context of gastrointestinal motility and the biochemical processes that influence it. The blue cluster’s keywords encompass “chronic constipation,” “dietary fiber,” “functional constipation,” “gastrointestinal symptoms,” “gut microbiome,” “microbiota,” and “probiotics.” This collection of terms suggests a focused research area that explores the role of the gut microbiome in FC, the impact of dietary interventions, particularly fiber and probiotics, on gastrointestinal health, and the broader implications for gastrointestinal symptoms management. The purple cluster’s keywords, including “depression,” “fecal microbiota transplantation,” “intestinal microbiota,” and “slow transit constipation,” point to a concentrated area of research within the field. This area of focus examines the psychological dimension of gastrointestinal health, with an emphasis on the exploration of depression in conjunction with gut-related issues.

Figure 8B presents an overlay visualization of the keywords, where the temporal distribution is indicated by color intensity. Keywords that have emerged more recently are depicted in shades of yellow. The analysis of the temporal trends reveals that “microbiota,” “probiotics,” and “dysbiosis” were predominant themes in the initial phases of the research. However, a shift is observed in more recent years, with research hotspots converging on terms like “gut microbiota,” “intestinal flora,” and “gastrointestinal motility,” suggesting an evolution in the focus areas of the field.

Figure 8D illustrates the chronological shift in the prevalence of keywords linked to GM and FC between 2013 and 2024. The visual representation captures the evolving research priorities within the scientific community. Circle size is indicative of keyword frequency, and line length denotes the period of significance for each term. Prominently, the analysis underscores “constipation,” “gut microbiota,” “intestinal motility,” and “gastrointestinal motility” as dominant themes, reflecting contemporary research foci and emerging trends.

### 3.8 Thematic map analysis

Thematic mapping, a technique derived from co-word analysis, is crucial for tracking the evolution of concepts across various research areas. It identifies thematic clusters that represent overarching research themes. Within this map, centrality is depicted along the horizontal axis, indicating how integral a theme is to the field, while the vertical axis represents density, which signifies the theme’s level of development. A cluster’s density indicates its internal cohesion, and its centrality shows how interconnected it is with other clusters. Clusters with high density are indicative of mature themes, while those with high centrality suggest themes that are either influential or central to the field’s discourse.

The distribution of these clusters across quadrants visually captures the development and significance of themes, aiding researchers in pinpointing both emerging and established topics within their area of study.

**Figure 9:**
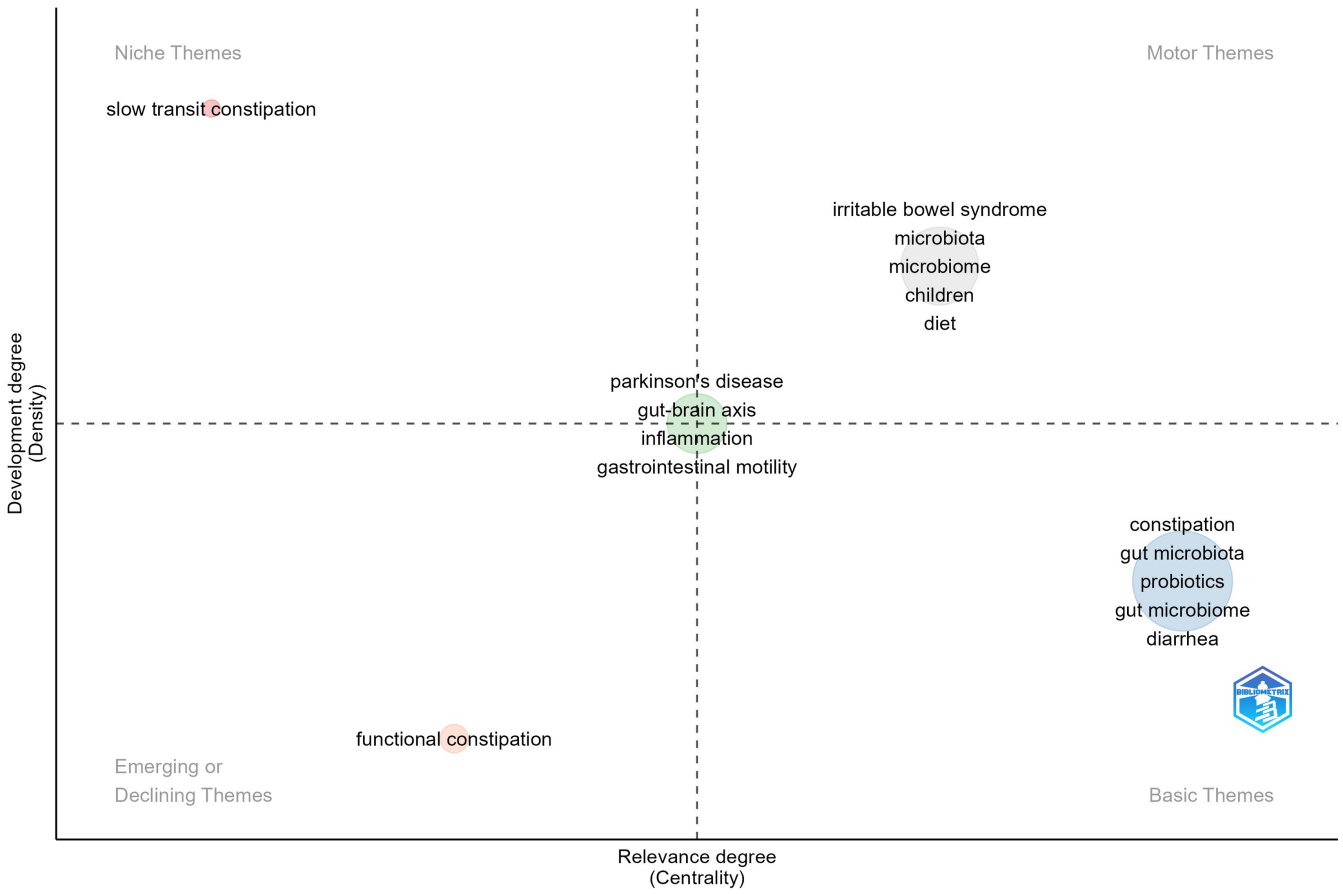
Thematic map outlining the research themes in GM and FC.

Figure 9 displays a thematic map outlining the research themes in GM and FC. The first quadrant, labeled “Motor Themes,” includes keywords such as “irritable bowel syndrome,” “microbiota,” “microbiome,” “children,” and “diet,” which are central and well-established in the field, reflecting extensive research and interconnections with other themes. The second quadrant, “Niche Themes,” features “slow transit constipation,” indicating a specialized area of study that, while developed, is less central to the overall research landscape. The third quadrant, “Emerging or Declining Themes,” contains “functional constipation,” suggesting a theme that is either gaining interest or experiencing a decline. Lastly, the fourth quadrant, “Basic Themes,” includes keywords like “constipation,” “gut microbiota,” “probiotics,” and “diarrhea,” which are recognized as important but require further exploration to fully understand their impact. Notably, themes such as “Parkinson’s disease,” “gut-brain axis,” “inflammation,” and “gastrointestinal motility” are positioned at the intersection of all four quadrants, highlighting their significance and potential for interdisciplinary research.

## 4 Discussion

FC is a widespread gastrointestinal disorder that impacts millions globally, transcending geographical and demographic boundaries, and is characterized by infrequent bowel movements and difficulty in defecation. Recent research has shed light on the significant role of GM in regulating gut motility, suggesting that dysbiosis, or an imbalance in these microbial communities, could contribute to the development of constipation. The burgeoning body of research articles and reviews dedicated to the study of GM and FC affirms the notable importance of these areas within the international scientific community. The swift pace of advancements, coupled with the global collaborative efforts evident in these publications, is instrumental in achieving a comprehensive understanding of the subject matter. Such an understanding is crucial for promoting effective collaboration and facilitating the exchange of knowledge among researchers from a variety of academic and professional disciplines.

### 4.1 Overview of research between GM and FC

This study presents a bibliometric analysis of the literature on GM and FC in the WoSCC. It examines the research landscape, including publication trends, geographical and institutional distributions, author collaborations, journal analyses, co-citation references, and keyword trends, to provide a concise overview of the field’s current status and collaborative aspects. From 2013 to 2024, this study has encompassed a total of 1,405 papers, indicating a notable and consistent rise in both the volume of publications and the number of citations received annually. The annual growth rate of publications was 18.75%, with a production curve that suggests a continued upward trend, indicating sustained scholarly interest in the field. This trend underscores the sustained interest and growing importance of research focused on GM and FC.

The dataset reveals a pronounced geographic distribution in the research output pertaining to the GM and FC, with China asserting a dominant position. China’s scholarly contributions are noteworthy, having published 531 articles constituting 37.8% of the global total in this area of study. Regarding collaborative efforts, a robust collaboration is observed among China, the United States, and the UK, signifying a formidable research alliance. Such collaborative endeavors are essential for enhancing knowledge and deepening the comprehension of GM’s role in FC.

The dataset underscores Jiangnan University as a pivotal institution in the research on the interplay between GM and FC, with a notable publication count of 131 articles. This is followed by Harvard University, which contributes significantly with 81 articles, and the University of California System with 74 articles. These institutions are at the vanguard of this research domain, indicating a robust and well-established research focus in these areas. Jiangnan University’s research has made strides in understanding FC through the examination of the gut microbiome and its metabolic byproducts. A study revealed that patients with FC have a distinct gut microbiome composition, with higher levels of Bacteroides and butyrate-producing bacteria, and lower serum levels of arginine biosynthesis intermediates, suggesting potential diagnostic markers (Wang et al., 2023). A clinical trial demonstrated that *Bifidobacterium bifidum* CCFM16 can improve stool consistency and increase bowel movement frequency by modulating the GM and SCFA metabolism, with Clostridia playing a crucial role (Wang et al., 2022). Additionally, resistant starch intake has been linked to a shift in gut microbial diversity and function, promoting certain beneficial bacterial genera and enhancing key metabolic pathways (Chen et al., 2023). There is less interaction between institutions of different countries, with research collaboration often occurring within the same country, reflects a common pattern in international research collaboration. This trend may be attributed to various factors such as geographical proximity, shared language and culture, and the ease of communication and coordination within a national framework.

The author map reveals the key authors and their collaborative networks, indicating a widespread research interest in GM and FC across 56 countries, highlighting the field’s global collaborative nature. The bibliometric analysis reveals CHEN W and ZHANG H as prominent researchers in the field of GM and FC, with 30 and 27 articles, respectively. CHEN W also leads in total citations (863) and has the highest H-index (15). Professor Chen Wei from Jiangnan University is a distinguished researcher in the field of food science, particularly recognized for his significant contributions to the study of the GM and its relationship with FC (Zhang et al., 2021). Chen Wei’s studies highlight the significant role of GM in FC and have pioneered new paths for treating and comprehending this condition. His research on probiotics, particularly *Bifidobacterium longum*, has demonstrated potential in alleviating constipation by enhancing intestinal motility and adjusting the gut microbiota, as evidenced by relevant studies (Zhang et al., 2023). Additionally, his team has investigated how dietary modifications affect the GM composition and subsequently influence constipation (Wang et al., 2023). Collectively, these efforts have set the stage for the development of innovative therapeutic approaches and tailored health assessments, which are crucial for effectively managing FC.

Journal analysis plays a pivotal role in bibliometric studies, aiding researchers in identifying the most suitable journals for their work. Regarding publication output, the journals “Nutrients” (74 articles), “Food and Function” (35 articles), and “Frontiers in Microbiology” (32 articles) have been the most prolific in the field of GM and FC. “Nutrients” has shown a consistent publication record from 2013 to 2024, while “Food and Function” and “Frontiers in Microbiology” have maintained a steady upward trajectory since 2017. This analysis underscores the importance of these journals as key publication venues in this scientific domain. Researchers aiming to submit manuscripts in the field of GM and FC should consider these journals as top priorities.

Analysis of the co-cited references showed that the top 3 cited publications were from Ohkusa T, Dimidi E, and Parthasarathy. “Gut Microbiota and Chronic Constipation: A Review and Update,” was published in Frontiers in Medicine by Toshifumi Ohkusa and colleagues in 2019. This review article provides an overview of the role of GM in chronic constipation, which includes FC and constipation-type irritable bowel syndrome (IBS-C) (Ohkusa et al., 2019). The review also examines various treatments such as probiotics, prebiotics, synbiotics, antibiotics, and fecal microbiota transplantation (FMT), which have shown efficacy in treating chronic constipation with few side effects. The evidence indicates that dysbiosis of the GM may contribute to constipation, and targeted treatments for this dysbiosis could be a new option for managing the condition, particularly for those who do not respond to conventional therapies.

### 4.2 Relationship between GM and FC

Compared to healthy individuals, patients with FC exhibit significant differences in the abundance and diversity of their GM. Zoppi et al. discovered through fecal culture methods that higher levels of *Clostridium* and *Bifidobacterium* are linked to constipation in children, indicating a GM imbalance associated with constipation (Zoppi et al., 1998). This dysbiosis may involve a reduction in the abundance of beneficial bacteria, such as *Bifidobacterium* and *Lactobacillus*, and an increase in potentially harmful bacteria (Weiss and Hennet, 2017; Xiao et al., 2020). As microbial sequencing technology matures, our understanding of the changes in the structure and function of the GM has grown significantly. Since 2014, the emerging technique of 16S rRNA sequencing has indeed catalyzed a surge in research on the GM. Led by Zhu, the research team found distinct GM imbalances in children with FC, implying that these microbial disruptions might affect gut function and exacerbate constipation symptoms (Zhu et al., 2014). The GM exerts a pivotal influence on FC. A primary mechanism by which the GM modulates FC is through the regulation of colonic motility, secretion, and absorption (Xiao et al., 2021). This regulation is facilitated by microbial metabolic activities such as short-chain fatty acid (SCFA) production, serotonin (5-HT), methane production, and bile acid (BA) biotransformation (Ohkusa et al., 2019; Martin-Gallausiaux et al., 2021; Buey et al., 2023). The balance of different BA molecules is crucial in regulating colonic motility (Lin et al., 2023). The unique role of the gut microbiota in transforming primary bile acids into secondary bile acids suggests a potential link between dysbiosis and colonic dysmotility (Cai et al., 2022). Additionally, SCFAs, as the predominant microbial fermentation products, have been shown to stimulate electrolyte absorption and suppress mucin secretion, contributing to the pathogenesis of FC (Martin-Gallausiaux et al., 2021). The impact of SCFAs on colonic motility is an active area of research, with evidence suggesting that SCFAs can affect the release of 5-HT from enterochromaffin (EC) cells (Lund et al., 2018; Gao et al., 2020). This 5-HT, in turn, can activate 5-HT3 and 5-HT4 receptors to stimulate colonic motility (Mawe and Hoffman, 2013). Furthermore, the GM is implicated in the regulation of 5-HT levels, which are known to play a role in the pathogenesis of FC (Agus et al., 2018). The generation of metabolites that modulate the synthesis, secretion, and functional activity of 5-HT in the intestinal environment is a key regulatory mechanism. These pathways significantly affect gastrointestinal motility, suggesting that targeting the GM’s metabolic activities could hold therapeutic potential for managing gastrointestinal disorders.

### 4.3 Emerging trends and future forecasts in GM and FC research

Keywords are essential for identifying the focal points and content of scholarly literature. Their prevalence within a field’s academic works can reveal current research hotspots. The visualization and clustering of keywords can also clarify the evolution of research topics over time. In the context of research related to the GM and FC, keywords such as “constipation,” “gut microbiota,” and “probiotic” have remained areas of intense interest. Since 2014, the research on the GM and its association with FC has seen a significant surge, driven by advancements in sequencing technology and a growing understanding of the microbiota’s role in gastrointestinal health. Ongoing research is concentrated on deciphering the complex interplay between the GM and constipation, with a particular emphasis on how microbial imbalances can modulate symptoms, transit times, and gas production (Pan et al., 2022). This area is anticipated to remain a central research priority, offering insights that could lead to novel therapeutic interventions for constipation. There is a burgeoning interest in examining the impact of various therapeutic approaches on the GM and their potential to alleviate constipation. This includes the study of probiotics and dietary interventions, which are projected to be significant research foci moving forward. Research has shown that probiotics can increase bowel movement frequency and reduce intestinal transit time in patients with FC (Dimidi et al., 2017). A systematic review and meta-analysis suggests that probiotic supplementation is moderately efficacious in decreasing intestinal transit times compared to control groups, indicating their potential for treating chronic idiopathic constipation (Garzon Mora and Jaramillo, 2024). The beneficial effects of probiotics are thought to be due to their ability to modulate both the composition and functionality of the gastrointestinal microbiota, thereby enhancing intestinal motility and influencing various biochemical factors. However, it’s important to note that the efficacy of probiotics can vary, and a meta-analysis has suggested that probiotics may not be effective in treating FC in children (Wallace et al., 2022). This difference in therapeutic outcome could be related to the distinct composition of the GM between adults and children (Milani et al., 2017). Dietary intervention is a significant area of research in the study of GM and its relationship with FC. Dietary interventions often focus on increasing the intake of dietary fibers, which are known to promote the growth of beneficial bacteria in the gut (Cotillard et al., 2013). A double-blinded randomized placebo-controlled trial evaluated the effects of dietary fibers or probiotics on FC symptoms and the modulation of GM (Lai et al., 2023). The study found that dietary fibers or probiotics may relieve hard stool, with intervention-specific changes in GM relevant to constipation relief. The study also suggested that baseline GM may predispose the intervention responsiveness. The influence of diet and lifestyle on the GM and its implications for FC are being increasingly recognized. High-fiber diets and other dietary interventions are being studied for their potential to modulate the GM and improve constipation. As research continues, we can expect further breakthroughs that will enhance our understanding of the GM’s role in FC and lead to new strategies for its management. The field is ripe for innovation, with potential for significant improvements in diagnostic methods, therapeutic interventions, and personalized treatment plans for patients with FC.

The gut-brain axis and inflammation are burgeoning areas of research in the context of GM and FC. The gut-brain axis encompasses the bidirectional communication between the central nervous system and the enteric nervous system (ENS) which is pivotal in regulating gastrointestinal function (Black et al., 2020). Within the scope of FC this axis is instrumental in modulating gut motility sensation and immune response (Margolis et al., 2021). The ENS often dubbed the “second brain” or “gut brain,” is a complex network of neurons and nerve fibers (Spencer and Hu, 2020). It is integral to the regulation of gastrointestinal function including the control of intestinal motility through the contraction and relaxation of smooth muscle (Uesaka et al., 2016). Emerging research underscores the ENS’s significant role in the etiology and progression of FC. The interplay between the GM and the ENS is complex and multifaceted. The GM can modulate ENS function by producing metabolic byproducts such as SCFAs (Dimidi et al., 2017). Furthermore shifts in the GM composition can impact the barrier function of intestinal epithelial cells potentially compromising ENS integrity (Dargenio et al., 2023). Enteric glial cells (EGCs) integral to the ENS are also involved in maintaining gut microbiota homeostasis (Vicentini et al., 2021). In the setting of FC dysbiosis of the GM and impaired EGC function may precipitate ENS dysfunction thereby disrupting intestinal motility and secretion (Erhardt et al., 2023)

Inflammation is a key player in the GM’s interaction with the host’s immune system (Cai et al., 2022). An imbalance in the gut GM, known as dysbiosis, can lead to low-grade inflammation, which has been associated with various gastrointestinal disorders, including FC (Di Rosa et al., 2023). This inflammation can affect the gut-brain axis, potentially leading to changes in gut motility and sensation, which are hallmarks of constipation (Banaszak et al., 2023). For instance, studies have shown that certain bacterial species are associated with constipation, and that these species may contribute to inflammation and altered gut-brain communication. Furthermore, the GM can influence inflammation through the metabolism of bile acids, production of SCFAs, and regulation of 5-HT levels—all of which can impact the gut-brain axis and contribute to constipation (He et al., 2020; Jia et al., 2018). The potential for dietary interventions to modulate the GM and, consequently, inflammation and gut-brain axis function is a promising area of research (Hess et al., 2021; Bordoni et al., 2017). By targeting the GM with specific diets, prebiotics, probiotics, or synbiotics, it may be possible to reduce inflammation, improve gut-brain communication, and alleviate constipation symptoms (Glassner et al., 2020). Understanding the complex interactions between the GM, inflammation, and the gut-brain axis could lead to new therapeutic strategies for managing FC.

Synthesizing the findings from the aforementioned analysis, future research in this domain is likely to exhibit the following trends: (1) Future studies will likely extend beyond FC to explore the role of GM in other constipation-related conditions or physiological processes. This holistic approach can provide a more comprehensive understanding of how GM influences various gastrointestinal disorder. (2) There is a growing interest in understanding how specific lifestyle factors, such as exercise and diet, modulate GM. Future research will delve deeper into the mechanisms by which these factors, along with pharmacological interventions, can reshape the GM to alleviate FC symptoms. (3) The application of new experimental techniques and detection markers in GM research will become more prevalent. These advancements will help researchers form more robust conclusions and enhance the credibility of their findings. (4) Probiotics, prebiotics, and synbiotics are being explored for their ability to regulate GM and improve FC. Future research will likely focus on identifying the most effective strains and formulations for treating FC.(5)The development of new therapeutic strategies targeting specific pathways influenced by GM, such as the gut-brain axis, is expected to be a significant area of future research.

## 5 Limitations

Our study acknowledges several limitations. Firstly, due to the challenges in directly integrating results from various databases and the limitations of scientometric software, we focused our search on the Web of Science (WoS) rather than combining it with results from other databases such as Scopus, PubMed, Embase, or MEDLINE. This decision means that our study may not encompass all journals, particularly those not indexed in WoS, including those that might be more thoroughly covered in MEDLINE, and it lacks representation from non-English journals, despite our emphasis on English-language publications. During the process of importing data into the bibliometric web application, there was an instance where some data was inadvertently lost. While our study offers a comprehensive look at the current state of research in our field, it is essential to consider these limitations, including the potential exclusion of relevant studies indexed in MEDLINE, when assessing the findings and when planning future research endeavors.

## 6 Conclusion

In our systematic study, we meticulously sorted and quantitatively analyzed the existing literature on GM and its relationship with FC. Our analysis sheds light on the current research landscape and delineates potential future trends within this specialized field. The findings from this research endeavor are designed to offer a holistic view of the current scientific discourse to researchers. Furthermore, it aims to serve as a valuable reference point for planning and guiding future investigative efforts in this domain. Drawing from our analysis, upcoming research on GM and FC is poised to expand its scope to other GI disorders, investigate the impact of lifestyle and pharmacological interventions on GM, adopt innovative techniques for robust findings, and explore probiotics, prebiotics, and synbiotics for FC treatment. Additionally, there’s a projected focus on developing therapies targeting GM-influenced pathways like the gut-brain axis.

## Data Availability

The original contributions presented in the study are included in the article/Supplementary material. Further inquiries can be directed to the corresponding authors.
