# Supplementary material for: Global research trajectories in gut microbiota and functional constipation: a bibliometric and visualization study
Source: Front Microbiol. 2024 Dec 6;15:1513723. doi: 10.3389/fmicb.2024.1513723 (PMC11659297; doi:10.3389/fmicb.2024.1513723)
Supplement: Supplementary file 1 [file Table_1.DOCX]

**Supplementary Material**

**Supplementary Table 1** Search Strategy

| Set | Results | Search Inquiry |
| --- | --- | --- |
| #1 | 25759 | TS=(functional constipation OR constipation OR Colonic Inertia OR Dyschezia) |
|  |  |  |
|  |  |  |
| #2 | 141573 | (((((((((((((((((((((((((((((((((((TS=(Gastrointestinal Microbiomes)) OR TS=(Microbiome, Gastrointestinal)) OR TS=(Gastrointestinal Microbial Community)) OR TS=(Gastrointestinal Microbial Communities)) OR TS=(Microbial Community, Gastrointestinal)) OR TS=(Gut Microbiome)) OR TS=(Gut Microbiomes)) OR TS=(Microbiome, Gut)) OR TS=(Gut Microflora)) OR TS=(Microflora, Gut)) OR TS=(Gastrointestinal Microflora)) OR TS=(Microflora, Gastrointestinal)) OR TS=(Gastrointestinal Flora)) OR TS=(Flora, Gastrointestinal)) OR TS=(Gut Flora)) OR TS=(Flora, Gut)) OR TS=(Gastrointestinal Microbiota)) OR TS=(Gastrointestinal Microbiotas)) OR TS=(Microbiota, Gastrointestinal)) OR TS=(Gut Microbiota)) OR TS=(Gut Microbiotas)) OR TS=(Microbiota, Gut)) OR TS=(Intestinal Microbiome)) OR TS=(Intestinal Microbiomes)) OR TS=(Microbiome, Intestinal)) OR TS=(Intestinal Flora)) OR TS=(Flora, Intestinal)) OR TS=(Intestinal Microbiota)) OR TS=(Intestinal Microbiotas)) OR TS=(Microbiota, Intestinal)) OR TS=(Intestinal Microflora)) OR TS=(Microflora, Intestinal)) OR TS=(Enteric Bacteria)) OR TS=(Bacteria, Enteric)) OR TS=(Gastric Microbiome)) OR TS=(Gastric Microbiomes) |
|  |  |  |
| #3 | 1405 | (#2 AND #1) AND (DT==("ARTICLE" OR "REVIEW") AND LA==("ENGLISH") AND PY==("2013" OR "2014" OR "2015" OR "2016" OR "2024" OR "2023" OR "2022" OR "2021" OR "2020" OR "2019" OR "2018" OR "2017") AND DT==("ARTICLE" OR "REVIEW")) |

**Supplementary Table 2** Top 10 countries of total global productions

| **Country** | **Articles** | **Articles %** | **SCP** | **MCP** | **MCP %** |
| --- | --- | --- | --- | --- | --- |
| CHINA | 531 | 37.8 | 478 | 53 | 10 |
| USA | 202 | 14.4 | 152 | 50 | 24.8 |
| JAPAN | 106 | 7.5 | 97 | 9 | 8.5 |
| ITALY | 73 | 5.2 | 55 | 18 | 24.7 |
| KOREA | 53 | 3.8 | 50 | 3 | 5.7 |
| UNITED KINGDOM | 43 | 3.1 | 25 | 18 | 41.9 |
| AUSTRALIA | 41 | 2.9 | 33 | 8 | 19.5 |
| POLAND | 31 | 2.2 | 28 | 3 | 9.7 |
| NETHERLANDS | 29 | 2.1 | 19 | 10 | 34.5 |
| INDIA | 22 | 1.6 | 19 | 3 | 13.6 |

Notes : SCP : single-country publications ; MCP : multiple-country publications.

**Supplementary Table 3** Annual publication output of the top 10 authors

| **Author** | **h_index** | **g_index** | **m_index** | **TC** | **NP** |
| --- | --- | --- | --- | --- | --- |
| CHEN W | 15 | 29 | 1.875 | 863 | 30 |
| ZHAO JX | 15 | 25 | 1.875 | 829 | 25 |
| ZHANG H | 14 | 27 | 1.75 | 778 | 27 |
| PIMENTEL M | 12 | 12 | 1.2 | 1052 | 12 |
| WANG G | 12 | 18 | 1.5 | 635 | 18 |
| WANG LL | 12 | 24 | 1.5 | 642 | 24 |
| LI N | 11 | 15 | 1.1 | 614 | 15 |
| ZHANG Y | 10 | 19 | 1.667 | 410 | 19 |
| CHEN J | 9 | 12 | 1 | 451 | 12 |
| SCHEPERJANS F | 9 | 10 | 0.9 | 1827 | 10 |

Notes : TC : Total citation; NP : Number of Publications

**Supplementary Table 4** Top 10 institution with the most significant contributions

| **Affiliation** | **Articles** |
| --- | --- |
| JIANGNAN UNIVERSITY | 131 |
| HARVARD UNIVERSITY | 81 |
| UNIVERSITY OF CALIFORNIA SYSTEM | 74 |
| MAYO CLINIC | 61 |
| UNIVERSITY OF LONDON | 55 |
| CAPITAL MEDICAL UNIVERSITY | 41 |
| UNIVERSITY OF HELSINKI | 41 |
| UNIVERSITY SYSTEM OF OHIO | 41 |
| CHENGDU UNIVERSITY OF TRADITIONAL CHINESE MEDICINE | 35 |
| NANJING UNIVERSITY OF CHINESE MEDICINE | 34 |

**Supplementary Table 5** Top 10 Journals with the most significant contributions.

| **Source** | **h_index** | **TC** | **NP** |
| --- | --- | --- | --- |
| NUTRIENTS | 19 | 1194 | 74 |
| FOOD & FUNCTION | 17 | 835 | 35 |
| FRONTIERS IN MICROBIOLOGY | 11 | 598 | 32 |
| SCIENTIFIC REPORTS | 12 | 1020 | 27 |
| INTERNATIONAL JOURNAL OF MOLECULAR SCIENCES | 9 | 453 | 25 |
| FRONTIERS IN CELLULAR AND INFECTION MICROBIOLOGY | 11 | 452 | 24 |
| NEUROGASTROENTEROLOGY AND MOTILITY | 12 | 601 | 23 |
| FRONTIERS IN NUTRITION | 9 | 241 | 22 |
| WORLD JOURNAL OF GASTROENTEROLOGY | 15 | 656 | 19 |
| DIGESTIVE DISEASES AND SCIENCES | 13 | 575 | 19 |

Notes : TC : Total citation; NP : Number of Publications

Supplementary Table 6 Top 10 Journals in terms of total citations.

| **Source** | **TC** |
| --- | --- |
| GASTROENTEROLOGY | 2611 |
| MOVEMENT DISORDERS | 2211 |
| MICROBIOME | 1615 |
| NUTRIENTS | 1194 |
| GUT | 1170 |
| PLOS ONE | 1070 |
| PARKINSONISM & RELATED DISORDERS | 1056 |
| AMERICAN JOURNAL OF GASTROENTEROLOGY | 1048 |
| SCIENTIFIC REPORTS | 1020 |
| FOOD & FUNCTION | 835 |

Notes : TC : Total citations.

**Supplementary Table 7** Top 10 most cited references

| Rank | Count | Cited references | Title | Publication Years | Source |
| --- | --- | --- | --- | --- | --- |
| 1 | 111 | Ohkusa T, 2019, FRONT MED-LAUSANNE, V6, P0, DOI 10.3389/fmed.2019.00019 | Gut Microbiota and Chronic Constipation: A Review and Update | 2019 | Frontiers in Medicine |
| 2 | 67 | Dimidi E, 2017, ADV NUTR, V8, P484, DOI 10.3945/an.116.014407 | Mechanisms of Action of Probiotics and the Gastrointestinal Microbiota on Gut Motility and Constipation | 2017 | Advances in Nutrition |
| 3 | 64 | Parthasarathy G, 2016, GASTROENTEROLOGY, V150, P367, DOI 10.1053/j.gastro.2015.10.005 | Relationship Between Microbiota of the Colonic Mucosa vs Feces and Symptoms, Colonic Transit, and Methane Production in Female Patients With Chronic Constipation | 2016 | Gastroenterology |
| 4 | 60 | Vriesman MH, 2020, NAT REV GASTRO HEPAT, V17, P21, DOI 10.1038/s41575-019-0222-y | Management of functional constipation in children and adults | 2020 | Nature Reviews Gastroenterology & Hepatology |
| 5 | 57 | Bharucha AE, 2020, GASTROENTEROLOGY, V158, P1232, DOI 10.1053/j.gastro.2019.12.034 | Mechanisms, Evaluation, and Management of Chronic Constipation | 2020 | Gastroenterology |
| 6 | 54 | Zhu LX, 2014, PHYSIOL GENOMICS, V46, P679, DOI 10.1152/physiolgenomics.00082.2014 | Structural changes in the gut microbiome of constipated patients | 2014 | Physiological Genomics |
| 7 | 54 | Vandeputte D, 2016, GUT, V65, P57, DOI 10.1136/gutjnl-2015-309618 | Stool consistency is strongly associated with gut microbiota richness and composition, enterotypes and bacterial growth rates | 2016 | Gut |
| 8 | 46 | Bolyen E, 2019, NAT BIOTECHNOL, V37, P852, DOI 10.1038/s41587-019-0209-9 | Reproducible, interactive, scalable and extensible microbiome data science using QIIME 2 | 2019 | Nature Biotechnology |
| 9 | 43 | Sampson TR, 2016, CELL, V167, P1469, DOI 10.1016/j.cell.2016.11.018 | Gut Microbiota Regulate Motor Deficits and Neuroinflammation in a Model of Parkinson's Disease | 2016 | Cell |
| 10 | 43 | Cao HL, 2017, SCI REP-UK, V7, P0, DOI 10.1038/s41598-017-10835-8 | Dysbiosis contributes to chronic constipation development via regulation of serotonin transporter in the intestine | 2017 | Scientific Reports |

**Supplementary Table 8** Most frequent keywords

| **Words** | **Occurrences** |
| --- | --- |
| constipation | 370 |
| gut microbiota | 264 |
| probiotics | 166 |
| irritable bowel syndrome | 143 |
| microbiota | 117 |
| parkinson's disease | 94 |
| microbiome | 80 |
| gut microbiome | 59 |
| diarrhea | 55 |
| functional constipation | 51 |
